# Identification of seipin-linked factors that act as determinants of a lipid droplet subpopulation

**DOI:** 10.1083/jcb.201704122

**Published:** 2018-01-02

**Authors:** Michal Eisenberg-Bord, Muriel Mari, Uri Weill, Eden Rosenfeld-Gur, Ofer Moldavski, Inês G. Castro, Krishnakant G. Soni, Nofar Harpaz, Tim P. Levine, Anthony H. Futerman, Fulvio Reggiori, Vytas A. Bankaitis, Maya Schuldiner, Maria Bohnert

**Affiliations:** 1 Department of Molecular Genetics, Weizmann Institute of Science, Rehovot, Israel; 2 Department of Biomolecular Sciences, Weizmann Institute of Science, Rehovot, Israel; 3 Department of Cell Biology, University of Groningen, University Medical Center Groningen, Groningen, Netherlands; 4 Department of Molecular and Cellular Medicine, Texas A&M Health Science Center, College Station, TX; 5 UCL Institute of Ophthalmology, London, England, UK

## Abstract

Functional heterogeneity within the lipid droplet (LD) pool of a single cell has been observed, yet the underlying mechanisms remain enigmatic. Here, we report on identification of a specialized LD subpopulation characterized by a unique proteome and a defined geographical location at the nucleus–vacuole junction contact site. In search for factors determining identity of these LDs, we screened ∼6,000 yeast mutants for loss of targeting of the subpopulation marker Pdr16 and identified Ldo45 (LD organization protein of 45 kD) as a crucial targeting determinant. Ldo45 is the product of a splicing event connecting two adjacent genes (*YMR147W* and *YMR148W/OSW5/LDO16*). We show that Ldo proteins cooperate with the LD biogenesis component seipin and establish LD identity by defining positioning and surface-protein composition. Our studies suggest a mechanism to establish functional differentiation of organelles, opening the door to better understanding of metabolic decisions in cells.

## Abstract (precis)

Eisenberg-Bord et al. describe a lipid droplet (LD) subpopulation with a unique proteome, which is adjacent to the nucleus–vacuole junction contact site. They identify the LD machinery, which cooperates with the lipodystrophy factor seipin as a key determinant of LD identity and suggest a mechanism for functional organelle diversification.

## Introduction

Lipid droplets (LDs) are evolutionarily conserved lipid storage organelles (Gross and Silver, 2014). As such, they need to dynamically balance deposition and mobilization of diverse lipid species to sustain crucial cellular functions, including metabolic homeostasis and biosynthesis of membrane lipids (Farese and Walther, 2009; Welte, 2015; Schuldiner and Bohnert, 2017). Accordingly, LD dysfunction is connected to various pathologic conditions, including obesity, diabetes, steatohepatitis, and lipodystrophy (Greenberg et al., 2011; Walther and Farese, 2012). LDs consist of a central core of neutral storage lipids (mainly triacylglycerols and sterol esters) shielded from the aqueous cytosol by a phospholipid monolayer that accommodates numerous surface proteins (Thiam et al., 2013). Most LD surface proteins are enzymes involved in lipid metabolism, but the function of several LD proteins is still unknown (Athenstaedt et al., 1999; Binns et al., 2006; Grillitsch et al., 2011; Currie et al., 2014; Ohsaki et al., 2014). Intriguingly, in recent years, several cases have been reported in which specific LD surface proteins were found enriched or even exclusively localized on a fraction of LDs (Wolins et al., 2005, 2006; Krahmer et al., 2011; Hsieh et al., 2012; Wilfling et al., 2013; Ren et al., 2014; Moldavski et al., 2015; Zhang et al., 2016; Thiam and Beller, 2017). Similarly, various lipids have been found distributed unevenly among LDs (Rinia et al., 2008; Hsieh et al., 2012). These findings demonstrate that the LD pool within a single cell consists of distinct LD subpopulations and suggest that a functional differentiation of LDs might contribute to cellular lipid homeostasis. However, the molecular mechanisms that establish and maintain LD heterogeneity are currently unknown.

In *Saccharomyces cerevisiae* (from here on termed “yeast”), one protein that localizes preferentially to a subpopulation of LDs is the phosphatidylinositol transfer protein Pdr16 (Ren et al., 2014; Moldavski et al., 2015). In this study, we used systematic screening approaches to characterize the Pdr16-rich LD subpopulation and identified six additional proteins enriched on the same LDs. We show that two of those subpopulation residents, which we term Ldo45 (Ymr147w + Ymr148w) and Ldo16 (Ymr148w/Osw5), are the product of a unique splicing event of two overlapping genes and act as key determinants of LD identity. Ldo45 is crucial for targeting of Pdr16 to the LD subpopulation, and Ldo16 mediates accumulation of LDs in a unique niche in the cell, the nucleus–vacuole junction (NVJ) contact site, under conditions of nutrient deprivation. Ldo45 and Ldo16 interact with the seipin complex that controls LD composition. Indeed, overexpression of Ldo45 results in a generalized loss of LD identity similar to loss of function seipin mutants. Our results suggest that through localized modulation of seipin, Ldo proteins mediate LD differentiation.

## Results

### A unique LD subpopulation resides proximal to the NVJ

**Figure 1.**
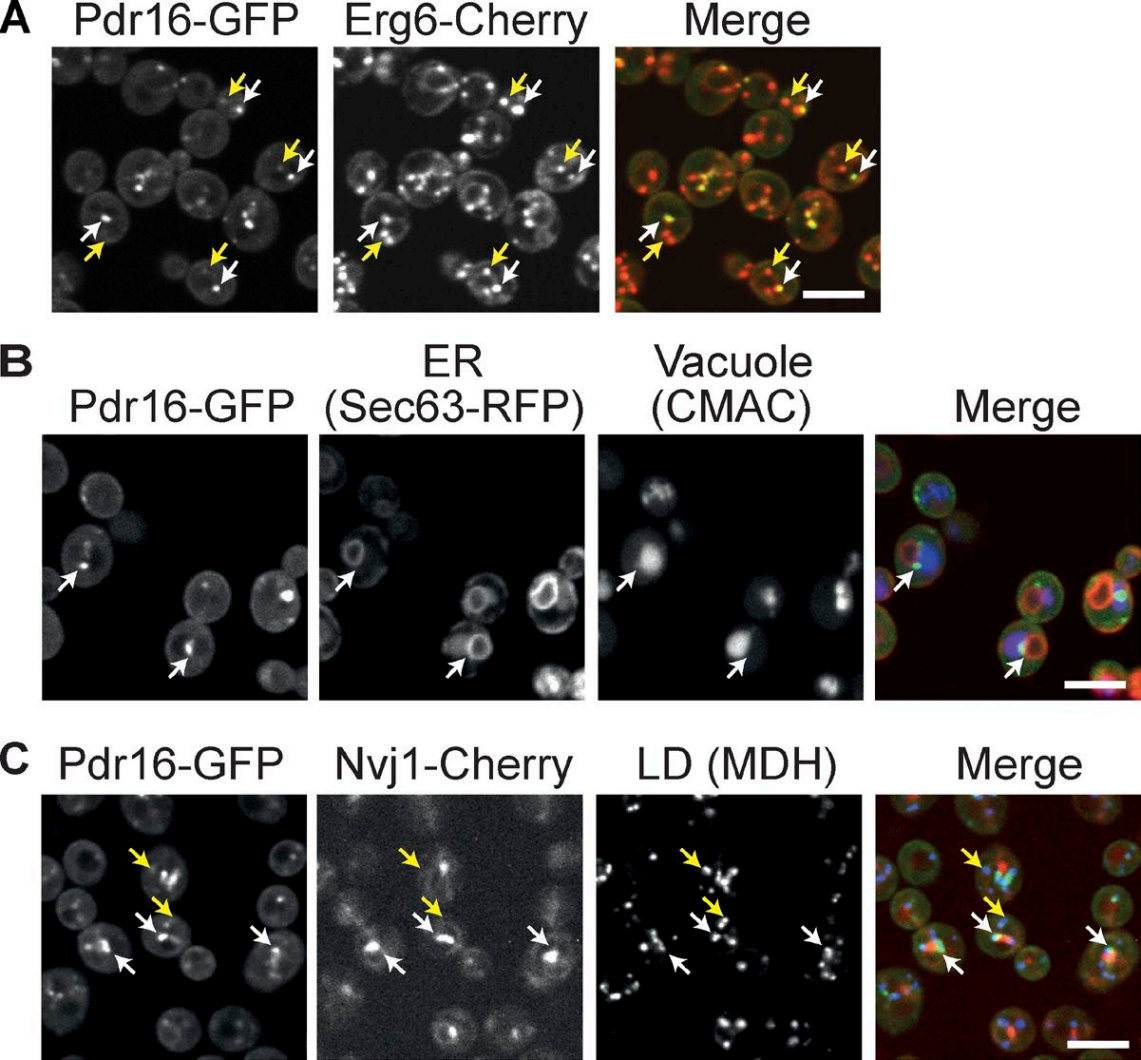
**A unique LD subpopulation located adjacent to the NVJ contact site. (A)** Pdr16-GFP marks a subset of Erg6-Cherry labeled LDs. White arrows, Pdr16-rich LDs; yellow arrows, Pdr16-poor LDs. Bar, 5 µm. **(B)** Coexpression of Pdr16-GFP and the ER marker Sec63-RFP and treatment with the vacuole dye CMAC revealed that Pdr16-rich LDs are often located adjacent to an area in which the nucleus and the vacuole are close to each other. Bar, 5 µm. **(C)** The NVJ marker Nvj1 was tagged with Cherry in a strain expressing Pdr16-GFP. Before imaging, the LD dye MDH was added. Pdr16-rich LDs next to the NVJ are marked with white arrows, Pdr16-poor LDs dispersed throughout the cell by yellow arrows. Bar, 5 µm.

We have previously shown that the LD protein Pdr16 is strongly enriched on just a fraction of cellular LDs in exponentially growing yeast cells (Moldavski et al., 2015). Typically, Pdr16 can be found on one LD per cell or alternatively on few LDs that are often close to each other (Fig. 1 A). Pdr16 is part of the family of Sec14-like phosphatidyl inositol transfer proteins (Li et al., 2000; Schnabl et al., 2003; Ren et al., 2014). This class of proteins has previously been suggested to preferentially localize to organellar contact sites (Šimová et al., 2013; Moldavski et al., 2015; Selitrennik and Lev, 2016), which are specific cellular subdomains where the surfaces of two organelles are actively positioned directly adjacent to each other by tether proteins (Eisenberg-Bord et al., 2016). We therefore sought to determine whether the Pdr16-rich LDs are in close proximity to any other cellular membrane. To this end, we expressed GFP-labeled Pdr16 (Pdr16-GFP) in cells with the different cellular membranes labeled with RFP or Cherry (Fig. S1 A). Although we could not detect any specific spatial relationship between Pdr16-rich LDs and the plasma membrane, peroxisomes or mitochondria, we found that LDs marked by Pdr16 were closely associated with both vacuolar and ER/perinuclear membranes.

To visualize both organelles at the same time, we labeled vacuoles with the blue vacuole luminal dye 7-amino-4-chloromethylcoumarin (CMAC) in cells expressing Pdr16-GFP and the ER marker Sec63-RFP (Fig. 1 B). We found that indeed, Pdr16-rich LDs were often found adjacent to the area where the nucleus and the vacuole were in close proximity to each other, a contact site termed the NVJ (Pan et al., 2000). To test whether Pdr16-rich LDs have a defined spatial relationship to this structure, we genomically tagged the NVJ marker protein Nvj1 with Cherry and found that Pdr16-rich LDs were preferentially located adjacent to the NVJ, whereas Pdr16-poor LDs (labeled by the neutral lipid dye monodansylpentane [MDH]) were dispersed throughout the cell (Fig. 1 C and Fig. S1 B). We conclude that Pdr16-rich LDs are spatially confined to a specific cellular location next to the NVJ.

### A high-content screen uncovers modulators of Pdr16 localization

**Figure 2.**
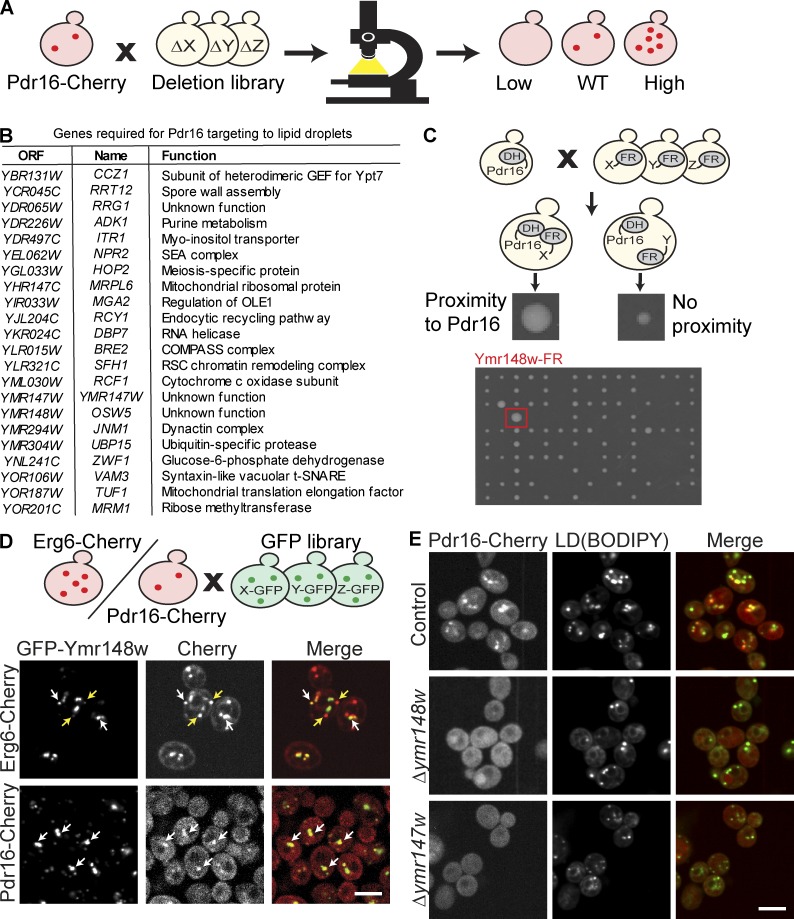
**High content screens identify localization modulators of the LD subpopulation marker Pdr16. (A)** Schematic representation of a visual systematic screen for factors involved in Pdr16 localization. A genome wide collection of ∼6,000 strains expressing Pdr16-Cherry on the background of single gene loss-of-function mutants was created. All strains were imaged by automated microscopy, followed by manual inspection to identify two classes of mutants with altered Pdr16 localization: mutants that fail to target Pdr16 to LDs (class “low,” see B for full list of hits), or mutants with more Pdr16-Cherry foci per cell (class “high,” see Table S1 for full list of hits). **(B)** List of mutants that fail to target Pdr16 to LDs (class “low”) from screen described in A. **(C)** Schematic representation of a whole-genome split-DHFR complementation screen. Pdr16 was tagged with one half of DHFR, and the resulting strain was crossed with a genome-wide library of mutants tagged with the second half of the enzyme. If Pdr16 and the second tagged protein are close to each other, the full-DHFR enzyme forms and cells grow to a bigger colony size compared with unrelated proteins. One hit was *YMR148W* (marked with a red square). A full list of hits can be found in Table S1. **(D)** Schematic representation of a visual colocalization screen for residents of the Pdr16-rich LD subpopulation. A strain expressing Pdr16-Cherry and a strain expressing Erg6-Cherry were crossed with a collection of strains expressing all known LD proteins tagged with GFP. GFP-Ymr148w was found enriched on a subset of Erg6-Cherry–positive LDs (white arrows), whereas other LDs showed only a weak GFP-Ymr148w signal (yellow arrows). Pdr16-Cherry and GFP-Ymr148w colocalized (white arrows). Bar, 5 µm. **(E)** Deletion of *YMR147W* or *YMR148W* results in the loss of targeting of Pdr16-Cherry to LDs detected by BODIPY. Bar, 5 µm.

An LD subpopulation that has both a defined local and a unique surface protein must have a molecular mechanism in place to determine its identity. To identify molecular determinants of this LD subpopulation, we used an unbiased systematic screen for factors involved in Pdr16 localization. We generated a genome-wide collection of ∼6,000 yeast strains expressing Pdr16-Cherry in the background of loss of function mutations (deletions for nonessential genes and decreased abundance by mRNA perturbation alleles for essential genes; Tong et al., 2001; Giaever et al., 2002; Tong and Boone, 2006; Breslow et al., 2008; Cohen and Schuldiner, 2011). All strains were imaged by automated microscopy, followed by manual inspection (Breker et al., 2013) to identify mutants with altered Pdr16 localization (Fig. 2 A). We identified 22 mutants that failed to target Pdr16 efficiently to LDs (Fig. 2 B) as well as 30 mutants with an increased number of Pdr16-positive foci compared with the control (Table S1).

Any one of the mutants that failed to properly target Pdr16 could be a direct determinant of LD identity or an indirect effector. To uncover the direct effectors, we performed two follow-up screens. First, we reasoned that a component involved in Pdr16 targeting should be found in proximity to Pdr16 and, thus, used a whole-genome, split dihydrofolate reductase (DHFR) complementation screen (Tarassov et al., 2008). In that screen, Pdr16 was tagged with one half of the DHFR enzyme and assayed for complementation with all other yeast proteins tagged with the other half, to search for components that are localized in the vicinity of Pdr16 (Fig. 2 C). We identified 39 hits in the split DHFR screen (Table S1). Second, we reasoned that factors required for Pdr16 targeting should be enriched on the Pdr16-rich subpopulation. To address that point, we assembled a collection of strains expressing all known LD proteins fused to either a C-terminal GFP moiety from the genome-wide C-terminal GFP library (Huh et al., 2003), or an N-terminal GFP tag under the control of a *NOP1* promoter from the SWAT GFP library (Yofe et al., 2016). We used an automated mating approach to cross each GFP-tagged strain with either a strain expressing Erg6-Cherry, which marks all LDs in the cell, or Pdr16-Cherry as a marker of our LD subpopulation. All strains were then imaged by automated microscopy and inspected manually. We identified five additional proteins that were enriched on the Pdr16-rich LD subpopulation (Fig. 2 D, Fig. S1 C, and Table S1): the lipid metabolism enzymes Erg2, Tgl4, and Srt1; and Ymr148w/Osw5 and Bsc2, two proteins of unknown function. We concluded that the Pdr16-rich LD subpopulation has both a unique cellular distribution and is equipped with a special set of proteins.

When cross-comparing the hits from all three screens (the primary Pdr16 targeting screen, the split DHFR screen, and the colocalization screen), we found that only one gene, *YMR148W/OSW5*, encoding a protein of unknown function, was identified by all three approaches. Ymr148w is thus enriched on Pdr16-rich LDs, can be found in close proximity to the Pdr16 protein, and in its absence, Pdr16 is no longer targeted to LDs. We, therefore, considered Ymr148w a highly promising candidate for a direct determinant of Pdr16 targeting.

Interestingly, *YMR147W*, the gene just upstream of *YMR148W*, was also a hit in the Pdr16-targeting screen. Manual recreation of the strains Pdr16-Cherry Δ*ymr148w* and Pdr16-Cherry Δ*ymr147w* showed that, although LDs were still present in both mutants, as confirmed by labeling with the neutral lipid dye boron-dipyrromethene (BODIPY) 493/503, both deletion of *YMR148W* and *YMR147W* led to a complete loss of Pdr16 targeting to LDs (Fig. 2 E).

### A unique splicing isoform is required for Pdr16 targeting to LDs

**Figure 3.**
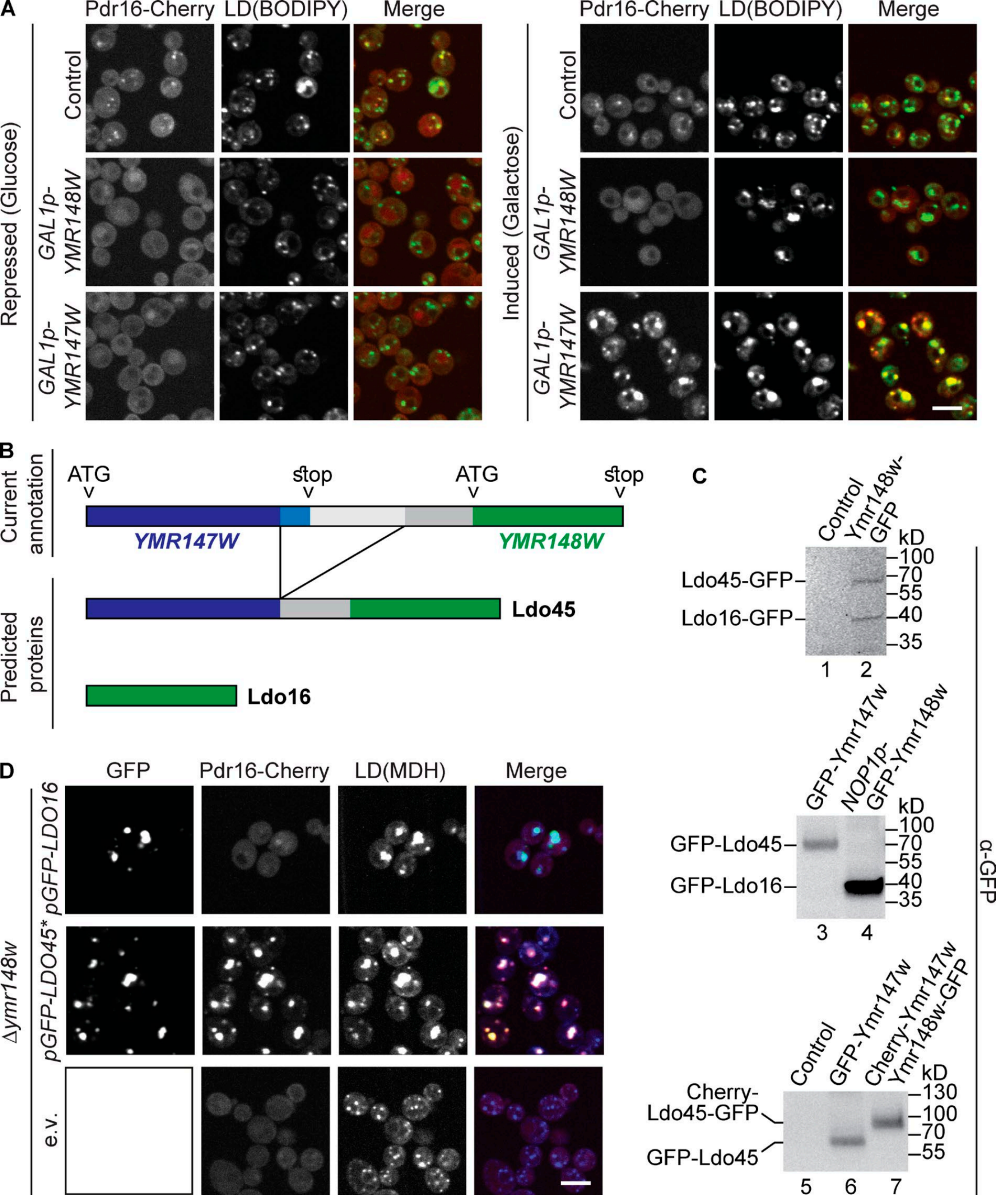
**Ldo45, the product of an intergenic splicing event, is required for Pdr16 targeting to LDs. (A)** A *GAL1* promoter was genomically integrated 5′ to *YMR147W* or *YMR148W* and targeting of Pdr16-Cherry to LDs, visualized with BODIPY, was assessed by fluorescence microscopy. Pdr16-Cherry targeting was abolished in the presence of glucose (repression) in both strains but rescued by incubation with galactose (induction) for 4 h only in *GAL1p-YMR147W* cells. Bar, 5 µm. **(B)** Schematic representation of the *YMR147W* (blue) and *YMR148W* (green) loci. A splicing reaction gives rise to a *YMR147W*-*YMR148W* fusion transcript (Miura et al., 2006) encoding Ldo45. That transcript corresponds to most of the *YMR147W* sequence (dark blue) excluding the last 90 nucleotides (light blue), 210 nucleotides of the annotated *YMR148W* promoter (dark gray), and the full *YMR148W* sequence (green). Ldo16 is the translation product of *YMR148W*. **(C)** Proteins from indicated cells were extracted and subjected to SDS-PAGE and Western blotting using anti–GFP antibodies. C-terminal GFP-tagging of *YMR148W* resulted in two bands corresponding to Ldo16-GFP and Ldo45-GFP. N-terminal tagging of *YMR148W* or *YMR147W* gives rise to only one tagged protein (GFP-Ldo16 or GFP-Ldo45, respectively). Simultaneous tagging of *YMR147W* with Cherry and *YMR148W* with GFP gives rise to a Cherry-Ldo45-GFP protein migrating ∼30 kD higher than GFP-Ldo45. Promoters used included an endogenous promoter, lanes 2 and 3; *NOP1p*, lane 4; and *TEF2p*, lanes 6 and 7. **(D)** cDNA plasmids encoding GFP-Ldo16 or GFP-Ldo45* (asterisk marking deletion of the *YMR148W* start codon) under control of a strong constitutive *TEF2* promoter or empty vector (e.v.) were transformed into cells with a genomic *ymr148w* deletion (lacking both Ldo16 and Ldo45) and targeting of Pdr16-Cherry to LDs, visualized with MDH, was assessed by fluorescence microscopy. Ldo16 is dispensable for Pdr16 localization, but overexpression of both Ldo isoforms induces alterations of LD morphology. Bar, 5 µm.

*YMR148W* was our main candidate for a factor involved in Pdr16 targeting, and we wondered whether the genomic manipulation of the neighboring gene *YMR147W* was, in fact, affecting the expression of *YMR148W.* To determine which of the two genes was responsible for the Pdr16-targeting phenotype, we created a Pdr16-Cherry strain expressing *YMR148W* under the control of the inducible/repressible *GAL1* promoter (*GAL1p*), as well as a strain expressing *YMR147W* under that same promoter. In the presence of glucose (i.e., *GAL1p* repression), we observed complete loss of Pdr16 targeting to LDs in both strains (Fig. 3 A, left). Surprisingly, induction of *YMR148W* expression by addition of galactose failed to restore Pdr16 localization (Fig. 3 A, right), whereas Pdr16 was targeted efficiently to LDs in the *GAL1p*-*YMR147W* strain under the same conditions, pointing toward a direct role for *YMR147W* in Pdr16 targeting. Of note, we observed Pdr16 targeting to all LDs instead of a subpopulation under this condition, likely because the *GAL1* promoter is strongly induced in the presence of galactose (Fig. 3 A, right, bottom). Additionally, we observed alterations in LD morphology upon overexpression of both proteins (Fig. 3 A, right; see section Overexpression of Ldo proteins results in LD clustering at the NVJ).

Our rescue experiments were confusing because, on one hand, Ymr148w came up in our split DHFR screen and was enriched in the LD subpopulation, but, on the other hand, it appeared that it was, in fact, Ymr147w that was required for Pdr16 targeting. In databases, *YMR147W* was originally annotated as a gene encoding a putative protein of unknown function. Intriguingly, several transcriptome studies identified a *YMR147W* transcript that does not correspond to the annotated gene (Miura et al., 2006; Reiner-Benaim et al., 2014; Schreiber et al., 2015). Strikingly, in this transcript, *YMR147W* is spliced to its downstream gene, *YMR148W*. Reverse transcription experiments templated with a total polyA^+^ RNA fraction purified from our WT strain, coupled to DNA sequence analysis of the amplified cDNA product, confirmed expression of this spliced RNA (unpublished data). The spliced transcript encodes a putative protein corresponding to amino acids 1–194 of the annotated gene *YMR147W*, 70 amino acids corresponding to part of the annotated promoter of *YMR148W*, as well as the complete *YMR148W* sequence (148 amino acids; Fig. 3 B).

To test whether such a fusion protein of Ymr147w and Ymr148w is expressed in vivo, we analyzed a strain with a C-terminal GFP tag on Ymr148w by Western blot using anti–GFP antibodies (Fig. 3 C, lane 2). Indeed, we detected two bands: the upper band corresponded to the expected size of the putative Ymr147w–Ymr148w fusion protein carrying a GFP tag (72 kD) whereas the lower band corresponded to the expected size of regular Ymr148w-GFP (43 kD). Anti-GFP Western blot of a strain encoding an N-terminal GFP tag on *YMR147W* resulted in just the 72-kD band corresponding to a tagged version of the putative fusion protein, indicating that this large protein was the major Ymr147w variant, whereas a protein corresponding to just the *YMR147W* gene is not expressed in detectable amounts (Fig. 3 C, lane 3). N-terminal GFP tagging of *YMR148W* resulted in the expected 43 kD band (Fig. 3 C, lane 4). Finally, we N-terminally tagged *YMR147W* with Cherry in a Ymr148w*-*GFP strain and subjected the cells to anti–GFP Western blot. We detected a band migrating ∼30 kD higher than the putative GFP-Ymr147w-Ymr148w band (Fig. 3 C, lanes 6 and 7), a shift that corresponds to the size of Cherry. Hence, the detected protein must contain both the GFP tag (used for detection) and the Cherry tag (causing the altered migration), further corroborating that the fusion protein from this unique intergenic splicing reaction was indeed expressed in vivo. We, thus, decided to name Ymr147w Ldo45 for LD organization protein of 45 kD, according to the molecular weight of the spliced product. Accordingly, we suggest renaming Ymr148w/Osw5 as Ldo16 (according to the molecular weight of this protein; also see Teixeira et al. in this issue).

In light of these findings, it is apparent that Ldo45, not Ldo16, is crucial for Pdr16 targeting. Introduction of the *GAL1p* cassette 5′ to the *LDO16* gene leads to disruption of expression of the spliced form of *LDO45* (Fig. 3 B), resulting in loss of Pdr16-Cherry targeting, both under induction and repression conditions (Fig. 3 A). In contrast, galactose induction of the *GAL1p*-*YMR147W* strain, which leads to synthesis of Ldo45 (Fig. 3 B), resulted in rescue of Pdr16-Cherry targeting (Fig. 3 A).

We asked whether Ldo16 was dispensable for Pdr16 targeting or whether both Ldo16/45 splice variants were required. Because deletion of the *YMR148W* gene resulted in the absence of both Ldo16 and Ldo45, we constructed a cDNA plasmid encoding only GFP-Ldo45 under control of a constitutive *TEF2* promoter that would allow for individual expression of Ldo45. Importantly, the *LDO45* sequence included both the complete *LDO16* coding sequence and part of the *LDO16* promoter (Fig. 3 B). Therefore, we deleted the three bases corresponding to the start codon of *LDO16* to abolish expression of *LDO16* from the plasmid, resulting in a plasmid that expressed only *LDO45* (pGFP-LDO45*; Fig. 3 D). As a control, we constructed an analogous plasmid pGFP-*LDO16*. When we transformed these plasmids into Δ*ymr148* cells, we found that pGFP-LDO45*, but not pGFP-*LDO16*, fully rescued the targeting of Pdr16-Cherry, showing that Ldo45 is essential for recruiting Pdr16 to LDs, whereas Ldo16 is dispensable (Fig. 3 D; Teixeira et al., 2018).

### Overexpression of Ldo proteins results in LD clustering at the NVJ

Knowing that Ldo45, but not Ldo16, is required for targeting of Pdr16 to the LD subpopulation, we were curious to determine the function of Ldo16. Intriguingly, we consistently observed that overexpression of either Ldo variant led to alterations in LD morphology, with numerous cells displaying a large, multilobed, neutral lipid-dye–positive structure located in the center of the cell (Fig. 3, A and D). We, therefore, asked whether, next to its function in Pdr16 targeting, Ldo16/45 could have an additional role in determining the specific subcellular localization of the Pdr16-rich LD subpopulation, close to the NVJ.

**Figure 4.**
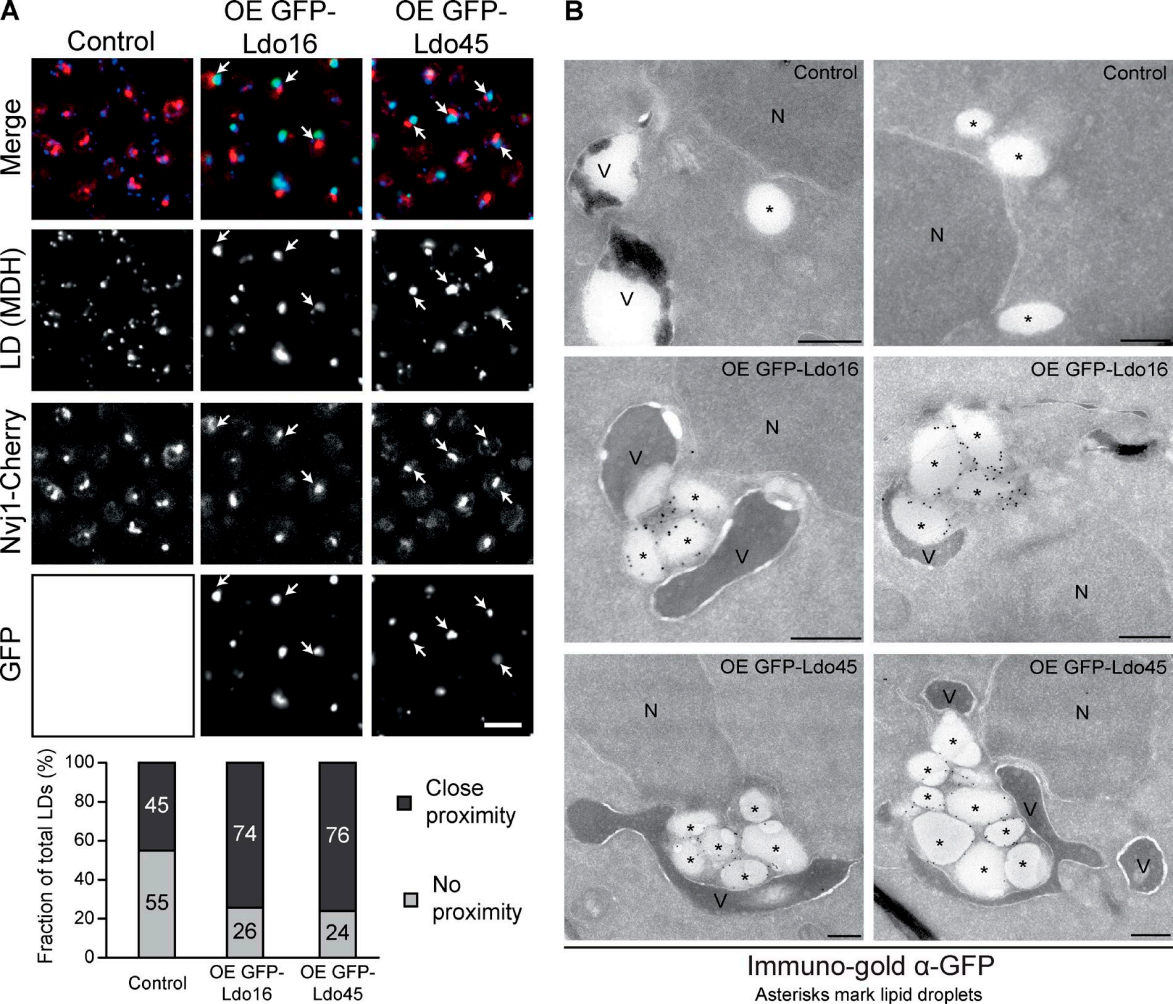
**Role of Ldo16 and Ldo45 in LD accumulation at the NVJ. (A)** Overexpression of GFP-Ldo45 and GFP-Ldo16 leads to accumulation of LDs next to the NVJ, marked by Nvj1-Cherry (arrows; top). This effect was quantified by counting the number of LDs labeled by MDH that were close to the NVJ (black) and LDs that were far from the NVJ (gray; bottom). *n* = 200 LDs. Bar, 5 µm. **(B)** Overexpression of GFP-Ldo45 or GFP-Ldo16 results in the formation of LD clusters adjacent to vacuolar and nuclear membranes, compared with Ymr148w-GFP control cells expressing Ldo proteins from their endogenous promoters, as determined by immunoelectron microscopy using anti–GFP antibodies. Asterisks mark LDs. N, nucleus; V, vacuole. Bar, 250 nm.

To assess a possible effect of Ldo16 or Ldo45 overexpression on the association of LDs with the NVJ, we integrated a *TEF2* promoter for constitutive overexpression of either GFP-Ldo16 or GFP-Ldo45 into an Nvj1-Cherry strain and stained the cells with the LD dye MDH. Similar to the phenotype observed upon acute induction of *GAL1p*-*YMR148W* and *GAL1p*-*YMR147W* (Fig. 3 A), constitutive overexpression of either Ldo isoform resulted in dramatic alterations of LD morphology with frequent appearance of a single, large, multilobed MDH-positive structure per cell, as opposed to approximately five small LDs per focal plane in control cells with unaltered Ldo16/45 levels (Fig. 4 A). Approximately 75% of the large MDH-positive structures in cells overexpressing Ldo16 or Ldo45 overlapped with the Nvj1-Cherry signal, as opposed to just 45% of control LDs (Fig. 4 A). These results prompted us to turn to immunoelectron microscopy to obtain high-resolution information on the exact nature of the multilobed structure as well as the spatial relationship between the NVJ and the altered LDs upon Ldo16/45 overexpression. Immunoelectron microscopy analysis revealed that the large MDH and BODIPY-positive structures in cells overexpressing Ldo proteins are clusters of several, tightly packed LDs, with Ldo45 overexpression resulting in larger LD clusters than Ldo16 produced. These LD clusters frequently appeared embraced from all sides by the nuclear and vacuolar membranes (Fig. 4 B). These results indicate synthetic overexpression of either Ldo protein is sufficient to relocate LDs to the NVJ.

### Ldo16 is a critical determinant for LD distribution during entry into stationary phase

**Figure 5.**
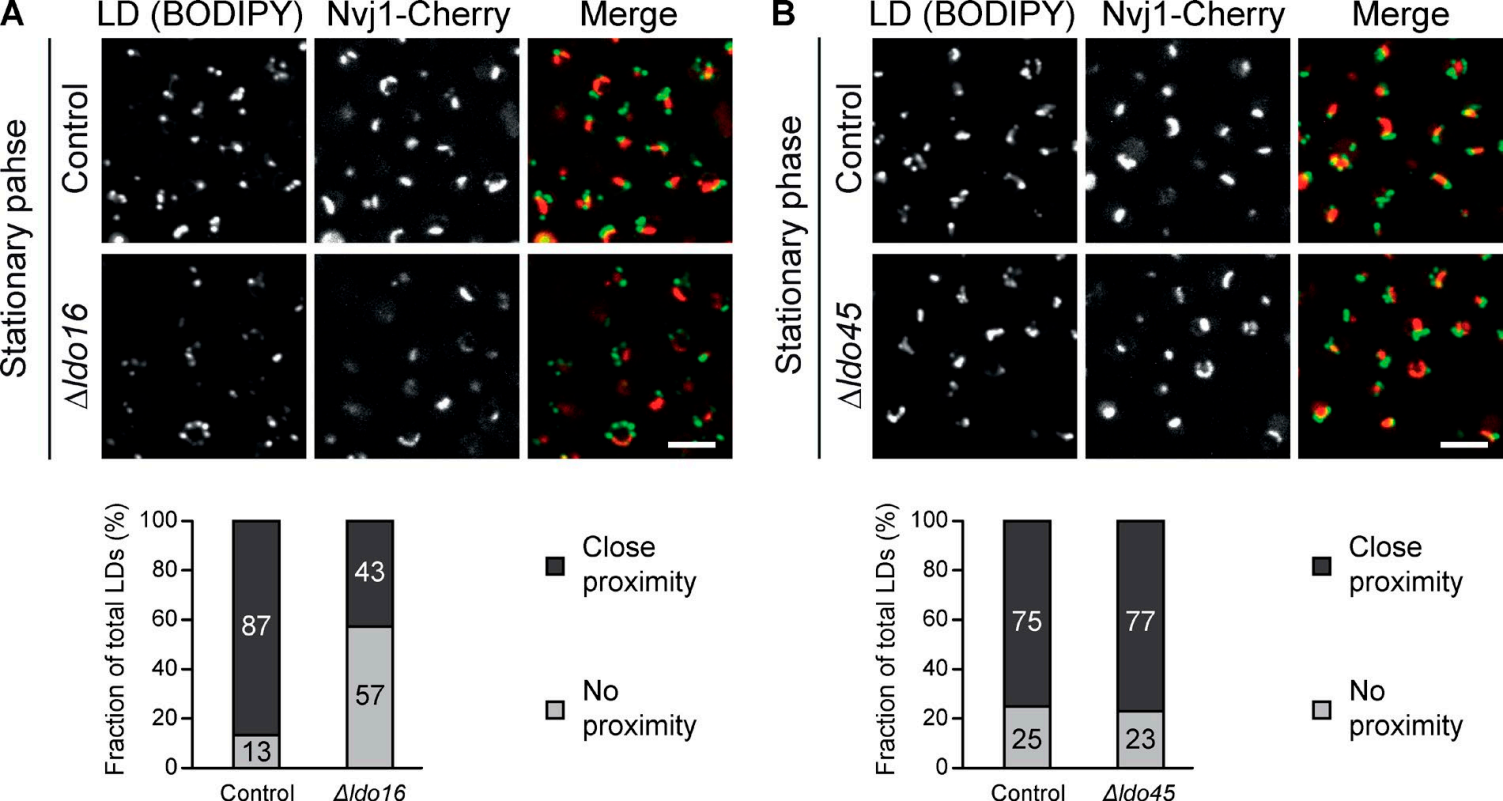
**Ldo16 is necessary for LD accumulation at the NVJ during entry into stationary-growth phase. (A)** Top: During entry into the stationary phase, LDs (labeled with BODIPY) accumulate at the NVJ (marked by Nvj1-Cherry) in control cells. This accumulation is markedly reduced in *Δldo16* cells, as quantified by counting the number of LDs labeled by BODIPY that were close to the NVJ (black) and LDs that were far from the NVJ (gray; bottom). *n* = 200 LDs. Bar, 5 µm. **(B)** Experiment performed as described in A using *Δldo45* cells shows that Ldo45 is dispensable for accumulation of LDs at the NVJ. *Δldo16* (A) and *Δldo45* (B) strains were generated in different background strains, resulting in a small difference in colocalization in the controls. *n* = 200 LDs. Bar, 5 µm.

We went ahead and searched for physiologic conditions under which LDs accumulate at the NVJ. In our standard experimental condition of exponentially growing culture, we usually find LDs dispersed throughout the cytosol; however, LDs have previously been found to accumulate at the NVJ once cells enter the stationary growth phase (Wang et al., 2014b; Barbosa et al., 2015; Fig. 5, A and B). Strikingly, we found that although 87% of WT LDs are in close proximity to the NVJ in stationary phase, only 43% of LDs in cells with a genomic deletion of *LDO16* were next to the NVJ under the same conditions (Fig. 5 A). This is virtually identical to the basal LD-NVJ colocalization determined in control cells in the exponential phase (45%; Fig. 4 A), indicating that, in Δ*ldo16* cells, LD accumulation at the NVJ during the diauxic shift was completely abolished. We found that deletion of *YMR147W*, which results in the absence of Ldo45, but normal expression of Ldo16, did not negatively affect LD accumulation at the NVJ (Fig. 5 B), indicating that, under physiologic conditions, Ldo16 is required for correct LD distribution, a function that can be fulfilled in the absence of Ldo45.

### A link between Ldo proteins and the LD biogenesis factor seipin

**Figure 6.**
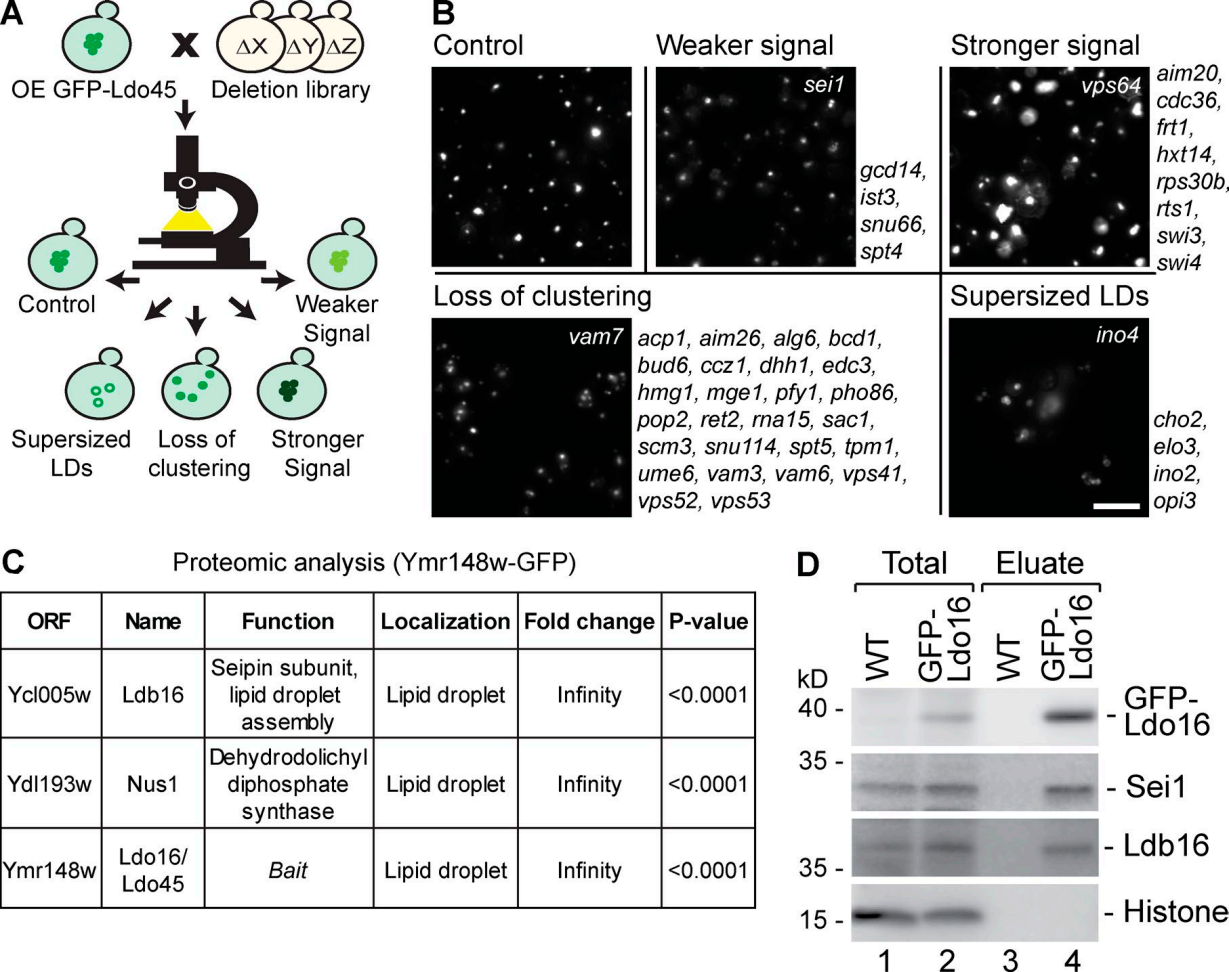
**Ldo proteins are linked to the LD biogenesis factor seipin. (A)** Schematic representation of a systematic screen for genes affecting the LD clustering phenotype observed in cells overexpressing Ldo45. A *TEF2p-GFP-LDO45* module for Ldo45 overexpression was introduced into the genome-wide deletion/decreased abundance by mRNA perturbation library and cells were visualized using automated microscopy. Phenotypes were categorized according to their trait: control phenotype with the typical LD clustering caused by Ldo45 overexpression; weaker GFP-Ldo45 signal; stronger GFP-Ldo45 signal; loss of the LD clustering typical for Ldo45 overexpressing cells; supersized LDs. **(B)** Representative images of all categories described in A. Loss-of-function mutation of the respective strains labeled in white; additional genes of the same category listed next to representative images. Bar, 5 µm. **(C)** Ymr148w-GFP cells (expressing both Ldo16-GFP and Ldo45-GFP) and untagged control cells were subjected to coimmunoprecipitation, followed by MS. Proteins that were enriched >2.4-fold and were statistically significant compared with control proteins are listed. **(D)** Cells expressing GFP-Ldo16 from a *NOP1* promoter were immunoprecipitated, and elutions were analyzed by SDS-PAGE and Western blotting.

We asked by which means Ldo16 and Ldo45 proteins exert their roles in defining LD identity. Because we could not detect significant alterations of the phospholipid composition between LD-enriched fractions from control, Δ*ldo16/45*, and Ldo16/45-overexpressing cells (Fig. S2 A), we reasoned that the Ldos might work through a proteinaceous machinery and thus searched for Ldo partner proteins. In a first approach, we performed a high-content screen for components required for the phenotype observed upon overexpression of Ldo45. We introduced a *pTEF2-GFP-LDO45* allele into the genome-wide deletion/decreased abundance by mRNA perturbation library and analyzed all strains by automated microscopy (Fig. 6 A). We identified several phenotypic classes (Fig. 6 B). (1) Strains that lost the LD clustering phenotype typical for Ldo45-overexpressing cells, resulting in reversion to a WT phenotype with LDs being dispersed throughout the cytosol; among these were components associated with splicing, the cytoskeleton, and nuclear and, especially, vacuolar membrane structure and dynamics; we speculate that in the splicing mutants, Ldo45 is not formed in sufficient amounts. (2) Several strains that had supersized LDs, all carrying mutations previously reported to induce supersized LDs by affecting phospholipid biosynthesis (Guo et al., 2008; Fei et al., 2011b). (3) Mutants with enhanced GFP signal, including genes involved in gene expression control. (4) Mutants displaying a weaker GFP-Ldo45 signal, comprising components required for splicing as well as the seipin component *SEI1* (Fig. 6 B). 

In a second approach, we performed coimmunoprecipitation of a Ymr148w-GFP strain (expressing both Ldo16-GFP and Ldo45-GFP; Fig. 3 B), followed by mass spectrometry (MS; Fig. 6 C). The top Ldo interactor was Ldb16, an ER protein that, alongside its partner protein Sei1, forms the seipin complex. In contrast, Ldb16 was not copurified from a strain expressing the highly abundant LD protein Erg6-GFP (unpublished data). In support of a link of seipin and LDO machineries, Ymr147w was previously identified by MS in coimmunoprecipitations using a GFP-tagged variant of Sei1, the second seipin component, as bait (Pagac et al., 2016). Furthermore, *SEI1* was also a hit in our visual screen for Ldo partner proteins, with *SEI1* deletion resulting in strongly reduced GFP-Ldo45 signal (Fig. 6 B). This phenotype is consistent with Sei1 being an Ldo interaction partner because loss of a physically interacting component often results in subsequent destabilization of the remaining partner protein. We reconfirmed the MS data by Western blotting and found that both Ldb16 and Sei1 were efficiently coisolated with GFP-tagged Ldo16 (Fig. 6 D, lane 4) as well as with Ldo45 (not depicted). Collectively, these results indicate that both Ldo16 and Ldo45 are linked to seipin (Teixeira et al., 2018).

### Antagonistic roles of seipin and LDO machineries

**Figure 7.**
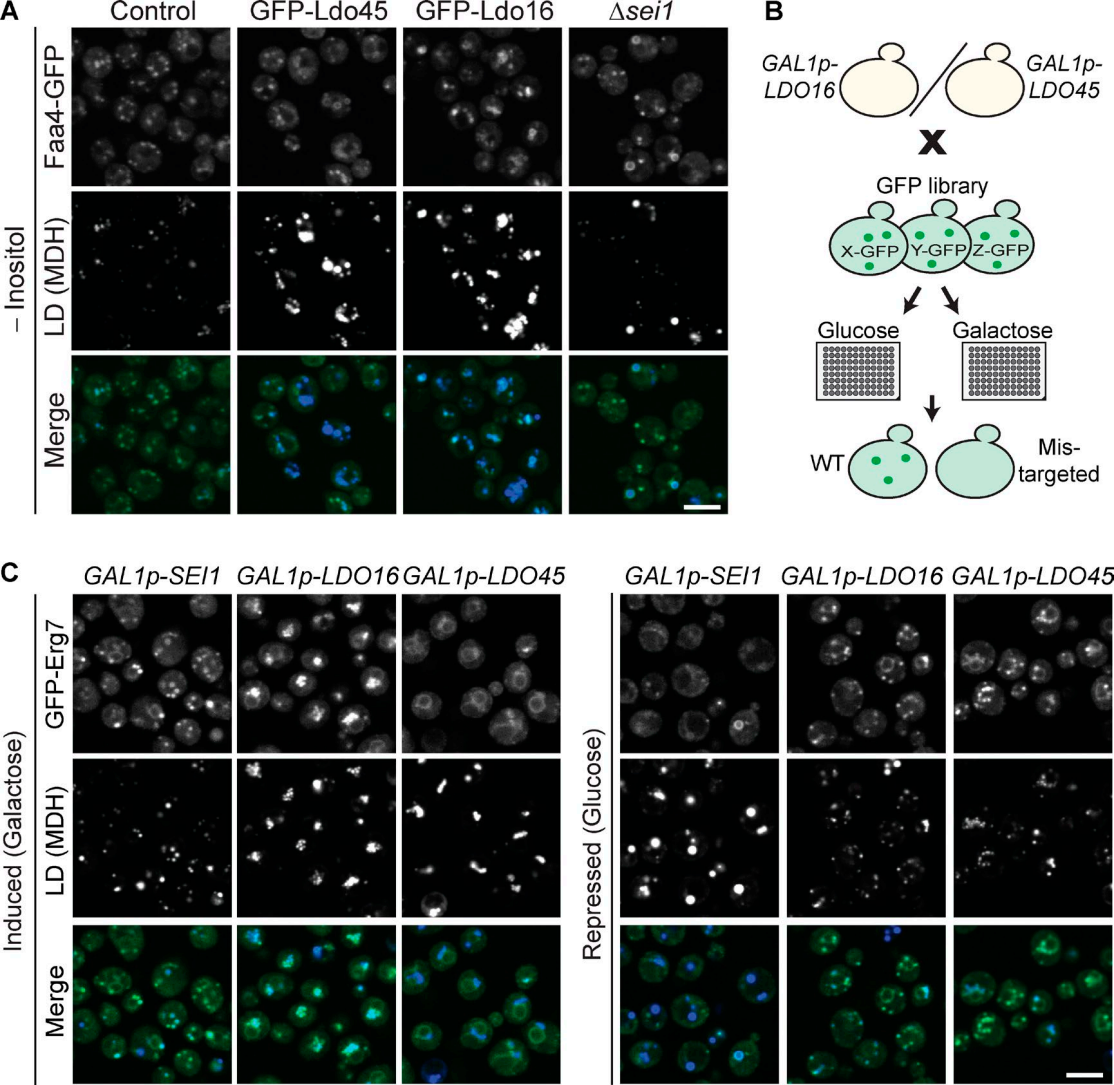
**Antagonistic roles of LDO and seipin machineries. (A)** Indicated cells were cultured in synthetic medium lacking inositol and analyzed by fluorescence microscopy. Supersized LDs in Δ*sei1* cells and in cells overexpressing Ldo proteins result in enlarged MDH signal as well as in the hollow appearance of the signal of the LD surface protein Faa4-GFP. Bar, 5 µm. **(B)** Schematic representation of a visual screen in which *GAL1p-LDO16* or *GAL1p-LDO45* cells were crossed with a library expressing all known LD proteins with an N-terminal GFP tag, grown on glucose (*GAL1p* repression) or galactose (*GAL1p* induction/overexpression) and imaged. 15 proteins were identified that failed to target efficiently to LDs exclusively upon Ldo45 overexpression. Full list of hits is provided in Table S1. **(C)** Example of targeting phenotype of a hit from the screen described in B. Strains GFP-Erg7 *GAL1p-LDO16* and GFP-Erg7 *GAL1p-LDO45* from the screen and a GFP-Erg7 *GAL1p-SEI1* control strain were imaged in the presence of glucose (repression) or galactose (overexpression). LDs were labeled with MDH. Upon overexpression of Ldo45 as well as repression of *SEI1*, Erg7 fails to localize to LDs. Bar, 5 µm.

Seipin is crucial for regular LD biogenesis and has been found mutated in patients suffering from Berardinelli-Seip congenital lipodystrophy (Magré et al., 2001). This disease has an intriguing combination of symptoms, presenting with virtually complete absence of subcutaneous adipose tissue, but with ectopic fat accumulation and a metabolic syndrome associated with high prevalence of diabetes (Agarwal and Garg, 2004). Despite the obvious importance of seipin in cellular biology and in human health, its exact molecular role is currently unclear. Two leading concepts suggest either a structural role of seipin in LD biogenesis or a role in regulation of phospholipid metabolism (Szymanski et al., 2007; Fei et al., 2008, 2011a; Cartwright and Goodman, 2012). Across species, seipin mutants generally display morphological alterations of LDs (Szymanski et al., 2007; Fei et al., 2008; Boutet et al., 2009). Yeast cells with deletions of *SEI1* or *LDB16* typically have aggregated LDs, similar to the morphology phenotype we observe upon overexpression of *LDO16* or *LDO45* (Fig. 4, A and B), suggesting that Ldo proteins could function in an antagonistic manner to seipin. To explore the idea of opposing phenotypes between the seipin complex and the LDO machinery, we turned to inositol-depletion conditions that lead to formation of supersized LDs in seipin mutants (Wang et al., 2014a). We tested whether that was also the case for Ldo overexpression and found that, indeed, overexpression of either Ldo protein resulted in formation of supersized LDs in inositol-depleted medium (Fig. 7 A).

Seipin has been detected at contact sites between LDs and the ER and has been suggested to have a direct or indirect role in protein sorting between those two organelles. A conserved feature from yeast to mammals seems to be that LDs are continuous with the ER membrane, likely via a phospholipid bridge (Jacquier et al., 2011; Wilfling et al., 2013). Many LD proteins, in particular those anchored to LDs via hydrophobic helical hairpins, are first inserted into the ER membrane and reach LDs via passage through such phospholipid bridges. It has recently been reported that, upon deletion of the seipin components, numerous LD proteins were not correctly targeted to LDs but were, instead, equally distributed between ER membranes and the LD surface, suggesting that the presence of seipin contributed to determination of the molecular identity of the LD surface as compared with the ER membrane (Grippa et al., 2015). To test whether alteration of Ldo16 or Ldo45 levels affects sorting of proteins from the ER to LDs, we introduced a *GAL1* promoter 5′ to *LDO16* or *LDO45* into our collection of strains expressing all known LD proteins with an N-terminal GFP tag by an automated approach and imaged all resulting strains in the presence of glucose (repressed *LDO16*/*LDO45*) or galactose (overexpressed *LDO16*/*LDO45*; Fig. 7 B). Intriguingly, we found that, exclusively upon overexpression of Ldo45, 15 bona fide LD proteins failed to efficiently accumulate on LDs. This phenotype resembled the protein-targeting defect observed in the absence of the seipin components Sei1 or Ldb16 (exemplified in Fig. 7 C and Fig. S2 B; full list of all 15 proteins affected is in Table S1; Teixeira et al., 2018). These mistargeted proteins likely contain helical hairpins and are thus expected to be targeted to LDs from the ER. These findings support the hypothesis that Ldo45 and seipin functions are antagonistic.

## Discussion

We report on the identification of a specialized LD subpopulation with a unique protein composition that is positioned next to the NVJ. Both characteristics are determined by the subpopulation residents Ldo16 and Ldo45, two proteins derived from overlapping genes with the latter formed by a unique, intergenic splicing event. Although Ldo45 is crucial for correct targeting of the subpopulation resident Pdr16, Ldo16 is involved in recruiting LDs to the NVJ.

Formation of subpopulations within the cellular LD pool might offer an expansion of the functional capacity of this organelle and/or enhance its flexibility in responding to environmental cues. How such heterogeneity of the cellular LD pool is established and maintained, however, is unknown. The morphology phenotype of Ldo16/45 overexpression strains as determined by fluorescence and immunoelectron microscopy shows that Ldo proteins induce clustering of LDs on vacuolar and ER membranes. Indeed, tethering to distinct partner organelles might be a simple and efficient way to induce LD heterogeneity by permanently locking them in specialized environments with unique features. At the same time, binding to a membrane surface generates a unique subdomain on the tethered LD that might attract specific proteins and/or lipids, the functions of which could further propagate into imprinting permanent molecular LD identity. Pdr16 is a lipid-transfer protein that has previously been suggested to function in the context of organelle contact sites and potentially can act as a tethering molecule (Šimová et al., 2013; Moldavski et al., 2015; Selitrennik and Lev, 2016). In addition, Pdr16 has been shown to inhibit fat mobilization from LDs via a phosphatidylinositol-4-phosphate–dependent mechanism (Ren et al., 2014), which is consistent with Pdr16 potentially acting as a rheostat on lipid exchange between the vacuole and LDs. Thus, we hypothesize that confinement to distinct cellular landmarks by tethering might be a general mechanism for imprinting LD identity and function. It is currently unclear whether Ldo16/45 directly act as molecular LD tethers or whether Ldo proteins promote formation of LD contact sites indirectly via downstream effectors. Candidates for downstream factors acting on LD recruitment to membrane surfaces are other proteins enriched in this unique LD subpopulation (Table S1) and components identified in our genome-wide screen for mutants affecting the LD clustering phenotype upon Ldo45 overexpression (Fig. 6 B).

One effector identified in this screen is seipin, an LD biogenesis component located at contact sites between LDs and the ER. Seipin has previously been reported to prevent equilibration of LD and ER surface components, thus affecting general LD identity as compared with the ER (Grippa et al., 2015). The interplay we find between Ldo45 and (to a lesser extent) Ldo16 with seipin may offer a clue to their function. Under physiologic conditions, Ldos are enriched on the NVJ specific LD subpopulation and hence may be modulating seipin activity only in specific LDs, resulting in altered entry of certain proteins and potentially enabling creation of an LD subpopulation with altered surface composition.

Seipin is conserved from yeast to human. According to basic primary sequence comparison, Ldo proteins are restricted to Saccharomycetes. Of note, one *Saccharomyces* strain has an *LDO45* gene corresponding to the spliced version of *S. cerevisiae LDO45* (GenBank: KOH48639.1), highlighting the functional importance of that component. Remote homology searches with HHsearch identify TMEM159/promethin as a protein with strong similarity to Ldo45 (Fig. S2 C). Promethin is found in numerous species, including human and fly. In fungi, occurrence of promethin is mutually exclusive with Ldo45, supporting an orthologous role for the two proteins. The closest proteins in terms of primary sequence to promethins are the oleosins, which are key structural LD proteins in plants (D’Andrea, 2016). Because promethin is up-regulated in liver cells during lipid storage conditions (Yu et al., 2004), and seipin has been identified as high-score interactor of promethin in a high-throughput affinity–purification MS approach (https://thebiogrid.org/166968/publication/the-bioplex-network-of-human-protein-interactions-additional-unpublished-ap-ms-results.html), we consider it a promising candidate for an Ldo homologue.

More broadly, by aiming to identify the molecular identity of a unique LD subpopulation and the mechanisms governing it, we have discovered a new LD protein formed by an intergenic splicing reaction and have elucidated a function for two previously uncharacterized proteins. Our results suggest a conserved mechanism for imprinting identity into organelles that underlies our bodies’ ability to maintain energy homeostasis.

## Materials and methods

### Strains and plasmids

*Saccharomyces cerevisiae* strains used in this study are described in Table S2, plasmids are described in Table S3. Plasmids Sec63-RFP-Ura and P4636-RFP-PTS1-URA were provided by J. Gerst (Weizmann Institute of Science, Rehovot, Israel). Plasmid pBS35-mCherry-hygromycin was provided by N. Barkai (Weizmann Institute of Science, Rehovot, Israel). Plasmid MTS-RFP-URA was provided by J. Nunnari (University of California, Davis, Davis, CA). Yeast strains were constructed from the laboratory strain BY4741 (Brachmann et al., 1998). Cells were genetically manipulated using a transformation method that includes the usage of Li-acetate, polyethylene glycol, and single-stranded DNA (Longtine et al., 1998; Janke et al., 2004; Gietz and Woods, 2006).

Primers for manipulations and validation were designed using Primers-4-Yeast (Yofe and Schuldiner, 2014).

### Yeast culturing and microscopy

Yeast cells were cultured overnight in synthetic minimal medium (0.67% [wt/vol] yeast nitrogen base with ammonium sulfate, 2% [wt/vol] glucose, amino acid supplements) at 30°C. Subsequently, cells were diluted and grown until reaching midlogarithmic phase. For experiments performed in the stationary phase, samples were kept undiluted. Cells were moved to glass-bottom, 384-well microscope plates (Matrical Bioscience) coated with Concanavalin A (Sigma-Aldrich). After 20 min, wells were washed twice with medium or with PBS to remove nonadherent cells. Different dyes were used for labeling organelles: For vacuolar lumen staining, CellTracker Blue CMAC Dye (10 µM; Thermo Fisher Scientific); for blue LD staining, MDH (100 µM; Abgent); and for green LD staining, BODIPY 493/503 (1 µM; Invitrogen).

Yeast cells were imaged at room temperature using a VisiScope Confocal Cell Explorer system composed of a Zeiss Yokogawa spinning disk scanning unit (CSU-W1) coupled with an inverted IX83 microscope (Olympus). Single–focal-plane images were acquired with a 60× oil lens (NA 1.4) and were captured using a PCO-Edge sCMOS camera, controlled by VisiView software (GFP [488 nm], RFP [561 nm], or BFP [405 nm]). Images were reviewed using ImageJ.

### Automated library preparation

Query strains for screens (Pdr16-Cherry, Erg6-Cherry, GFP-Ldo45, *GAL1p-LDO45*, and *GAL1p-LDO16*) were constructed on a synthetic genetic array ready strain and were integrated into yeast libraries using the synthetic genetic array method (Tong and Boone, 2006; Cohen and Schuldiner, 2011). A RoToR bench-top colony array instrument (Singer Instruments) was used to handle libraries (Tong and Boone, 2006; Cohen and Schuldiner, 2011). Strains from opposing mating types harboring the desired genomic manipulations (mutation/deletion/tag, etc.) were mated, and diploid cells were selected. Sporulation was induced (by moving the yeast to nitrogen starvation media for 7 d), and the haploid cells were selected using canavanine and thialysine (Sigma-Aldrich). By moving the haploid cells to plates containing selections for the combination of manipulations desired, a final library containing the genomic traits was created. Representative strains of the resulting screening libraries were validated by manual microscopy and check PCR.

### High-throughput microscopy

Libraries were screened at room temperature using an automated, inverted fluorescence microscopic ScanR system (Olympus), during midlogarithmic growth (Breker et al., 2013). Images were acquired using a 60× air lens (NA 0.9) with excitation at 490/20 nm (GFP) or 572/35 nm (RFP). After acquisition, images were manually reviewed using the ImageJ analysis program.

### Immunoelectron microscopy

Cells were fixed, embedded in gelatin, and cryosectioned as in Griffith et al. (2008). Sections were then immuno-labeled using rabbit anti–GFP (chromatin immunoprecipitation [ChIP] grade ab290; Abcam), followed by protein A–gold detection. Sections were imaged in a FEI CM100bio electron microscope at 80 KV, equipped with a digital camera (Morada; Olympus).

### Protein proximity assay (split-DHFR assay)

Pdr16 was C-terminally tagged with one half of a methotrexate-resistant variant of the essential DHFR enzyme, and the resulting strain was crossed with a library of strains in which each strain expressed one protein C-terminally tagged with the other half of the enzyme (Tarassov et al., 2008). Diploid cells were then moved to plates containing methotrexate, which inhibits the endogenous DHFR, whereas the mutated enzyme variant remains functional (Tarassov et al., 2008). Large colonies form if the tagged proteins are close to each other, allowing the formation of the mutated enzyme, whereas residual colonies remain if the tagged proteins are far apart. Colony size was analyzed using the Balony software (Young and Loewen, 2013).

### Preparation of whole cell extracts and Western blot

Cells expressing GFP tagged proteins and control cells were harvested by centrifugation, and proteins were extracted by NaOH or TCA extraction (Kushnirov, 2000; Ast et al., 2013). Samples were analyzed by SDS-PAGE and Western blotting using an anti–GFP antibody (ChIP grade ab290; Abcam). Membranes were either probed with a secondary antibody coupled to horseradish peroxidase (0545; Sigma-Aldrich) for visualization by enhanced chemiluminescence, or with a secondary antibody conjugated to IRDye800 (LI-COR Biosciences), followed by scanning using the Odyssey Imaging System.

### GFP affinity chromatography and MS

Cells expressing GFP-tagged proteins and untagged control cells were grown to midlogarithmic phase in synthetic minimal medium, harvested, washed in distilled water, and resuspended in lysis buffer (50 mM Tris HCl, pH 7, 150 mM NaCl, and protease inhibitors [complete EDTA-free cocktail]; Roche). Subsequently, samples were snap-frozen in liquid nitrogen and ground using a ball mill (30 s at 30 Hz; Retsch). Samples were resuspended in lysis buffer supplemented with 1% digitonin (Sigma-Aldrich) and incubated at 4°C for 1 h. After centrifugation at 19,000 rpm in an SW41 swing-out rotor for 30 min at 4°C, GFP-trap (Chromotek) was added, and samples were incubated for 1 h at 4°C. Three washes were performed with lysis buffer, followed by two additional washes in PBS.

For analysis by Western blot, bound proteins were eluted by the addition of 0.2 M glycine, pH 2.5. Samples were neutralized by the addition of 10% (vol/vol) 1 M Tris, pH 9.4, and analyzed by SDS-PAGE and Western blotting using an anti–GFP antibody (ChIP grade ab290; Abcam), and antibodies directed against Sei1, Ldb16 (both provided by P. Carvalho, University of Oxford, Oxford, England, UK), and histone H3 (ChIP grade ab1791; Abcam).

For analysis by MS, samples were subjected to in-solution, on-bead, tryptic digestion. 8 M urea in 0.1 M Tris, pH 7.9, was added onto PBS-washed beads and incubated for 15 min at room temperature. Proteins were reduced by incubation with dithiothreitol (5 mM; Sigma-Aldrich) for 60 min at room temperature, and alkylated with 10 mM iodoacetamide (Sigma-Aldrich) in the dark for 30 min at room temperature. Urea was diluted to 2 M with 50 mM ammonium bicarbonate. 250 ng trypsin (Promega) was added and incubated overnight at 37°C, followed by addition of 100 ng trypsin for 4 h at 37°C. Digestions were stopped by addition of trifluoroacetic acid (1% final concentration). After digestion, peptides were desalted using Oasis HLB μElution format (Waters), vacuum-dried, and stored at −80°C until further analysis. Ultra-liquid chromatography/MS-grade solvents were used for all chromatographic steps. Each sample was loaded using splitless nano-ultraperformance liquid chromatography (10 kpsi nanoAcquity; Waters). The mobile phase was a) H_2_O + 0.1% formic acid, and b) acetonitrile + 0.1% formic acid. Desalting of the samples was performed online using a reversed-phase Symmetry C18 trapping column (180 µm internal diameter, 20 mm length, 5 µm particle size; Waters). The peptides were then separated using a T3 high-strength silica nanocolumn (75 µm internal diameter, 250 mm length, 1.8 µm particle size; Waters) at 0.35 µl/min. Peptides were eluted from the column into the mass spectrometer using the following gradient: 4–20% b in 55 min, 20–90% b in 5 min, maintained at 90% for 5 min, and then back to initial conditions. The nano-ultraperformance liquid chromatography was coupled online through a nano-electrospray ionization emitter (10 µm tip; New Objective) to a quadrupole orbitrap mass spectrometer (Q Exactive HF; Thermo Scientific) using a FlexIon nanospray apparatus (Proxeon). Data were acquired in data-dependent acquisition mode, using a top-20 method. MS1 resolution was set to 120,000 (at 400 m/z), mass range of 300–1650 m/z, automatic gain control of 3e6, and maximum injection time was set to 20 msec. MS2 resolution was set to 30,000, quadrupole isolation 1.7 m/z, automatic gain control of 1e6, dynamic exclusion of 60 s, and maximum injection time of 60 ms. Raw data were imported into the Expressionist software version 10.5 (Genedata) and processed as described (Shalit et al., 2015). The software was used for retention-time alignment and peak detection of precursor peptides. A master peak list was generated from all MS/MS events and sent for database searching using Mascot v2.5.1 (Matrix Sciences). Data were searched against the *S. cerevisiae* sequences UniprotKB appended with common laboratory-contaminant proteins. Fixed modification was set to carbamidomethylation of cysteines, and variable modifications were set to oxidation of methionines and deamidation of N or Q. Search results were then filtered using the PeptideProphet algorithm (Keller et al., 2002) to achieve a maximum false-discovery rate of 1% at the protein level. Peptide identifications were imported back to Expressionist to annotate identified peaks. Quantification of proteins from the peptide data was performed using an in-house script (Shalit et al., 2015). Data were normalized based on the total ion current. Protein abundance was obtained by summing the three most-intense, unique peptides per protein. A two-sided Student’s *t* test, after logarithmic transformation, was used to identify significant differences across the biological replica. Fold changes were calculated based on the ratio of arithmetic means of the case versus control samples.

### Isolation of an LD-enriched fraction and phospholipid analysis by thin-layer chromatography

Cells were grown on minimal medium at 30°C overnight, back-diluted, and left to grow until they reached mid-logarithmic phase. Harvested cells were resuspended in DTT buffer (100 mM Tris-H_2_SO_4_, pH 9.4, and 10 mM DTT) and incubated for 20 min (30°C). Cells were collected by centrifugation and washed with Zymolyase buffer without enzyme (20 mM potassium-phosphate buffer, pH 7.4, and 1.2 M sorbitol). Subsequently, cells were resuspended in Zymolyase buffer and incubated in the presence of Zymolyase for 30 min. The enzyme was removed with a washing step using Zymolyase buffer without enzymes, and cells were resuspended in breaking buffer (10 mM Tris-HCl, pH 6.9, 0.2 mM EDTA, and 12% Ficoll 400) supplemented with 2 mM phenylmethylsulfonyl fluoride (Sigma-Aldrich). Cells were broken using a dounce homogenizer, and the resulting homogenate was subjected to a clarifying spin. Supernatants were adjusted to a volume of 6.7 ml using breaking buffer and transferred to ultracentrifugation tubes (331372; Beckman Coulter). Samples were overlaid with the same volume of breaking buffer and subjected to ultra-centrifugation in an SW41 swing-out rotor (28,000 rpm). The resulting white, floating layer enriched with LDs was collected using a bent glass pipette. Thin-layer chromatography analysis was performed on crude LDs using a developing solvent of chloroform/acetone/methanol/acetic acid/water (50:20:10:15:5, vol:vol:vol:vol:vol). Lipids were visualized using copper sulfate (6.25 mM in 9.4 ml phosphoric acid) at 110°C for 30–60 min. Bands were identified using phospholipid standards (Avanti Polar Lipids; Sigma-Aldrich).

### Online supplemental material

Fig. S1 characterizes the LD subpopulation located adjacent to the NVJ. Fig. S2 shows mechanistic aspects of LDO machinery function. Table S1 provides a list of hits from all screens performed in this study. In Table S2, all strains used in this study are described. Table S3 shows all plasmids used in this study.

## Supplementary Material

Supplemental Materials

Tables S1-S3 (Excel)
