# Supplementary material for: Identification of seipin-linked factors that act as determinants of a lipid droplet subpopulation
Source: J Cell Biol. 2018 Jan 2;217(1):269–82. doi: 10.1083/jcb.201704122 (PMC5748981; doi:10.1083/jcb.201704122)
Supplement: Supplemental Materials [file JCB_201704122_sm.pdf]

Eisenberg-Bord et al., https://doi.org/10.1083/jcb.201704122

THE JOURNAL OF CELL BIOLOGY

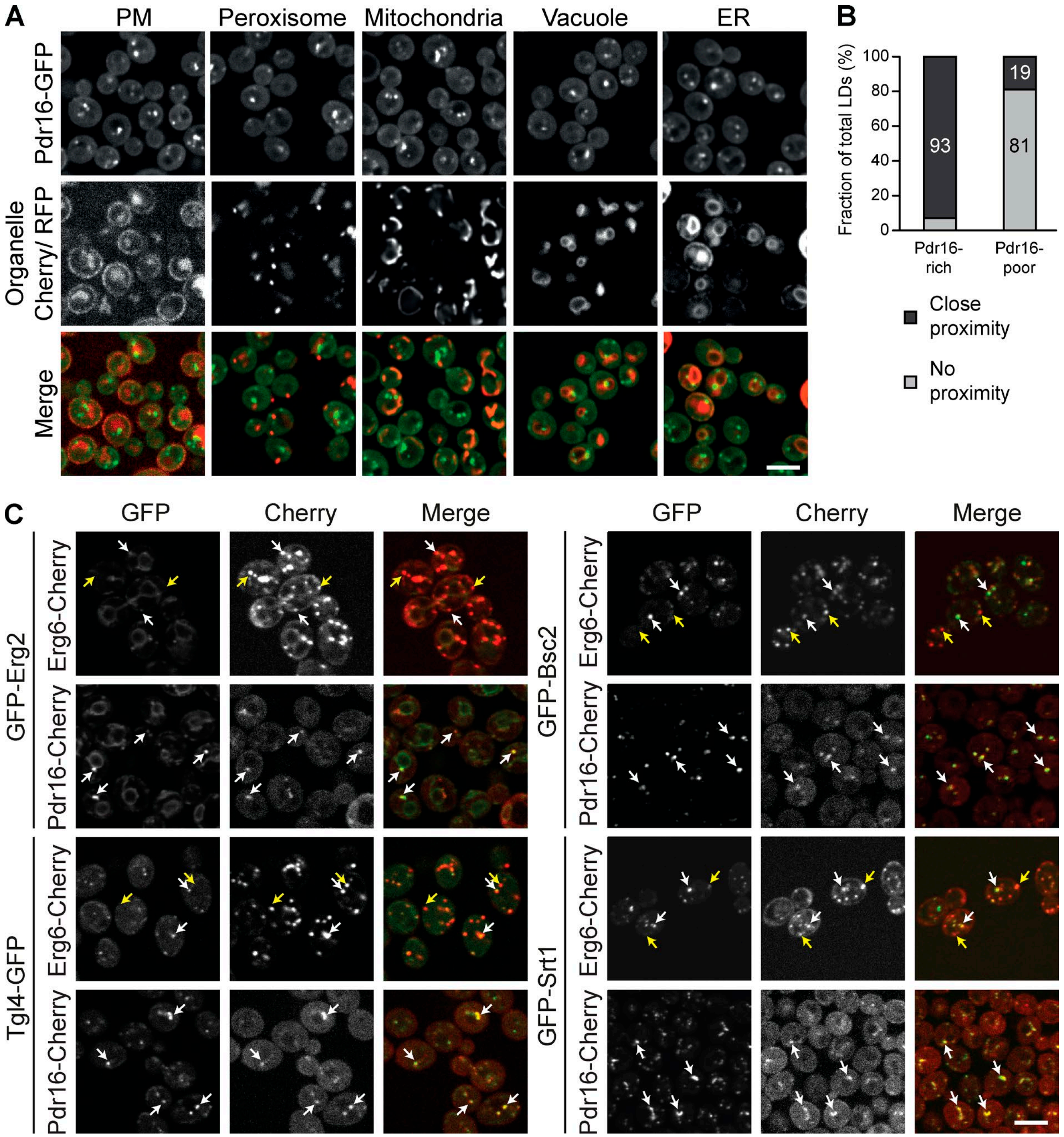

Figure S1. **An LD subpopulation with a unique protein composition is located adjacent to the NVJ. (A)** Pdr16-GFP was coexpressed with organelle markers: Mep3-Cherry for the plasma membrane (PM), RFP fused to a peroxisomal targeting sequence one for peroxisomes, RFP fused to a mitochondrial targeting sequence for mitochondria, Vph1-Cherry for the vacuole, and Sec63-RFP for the endoplasmic reticulum (ER). Pdr16-rich LDs are close to the vacuole and the nuclear ER. Bar, 5 µm. **(B)** Pdr16-rich LDs are preferentially located adjacent to the NVJ. The number of Pdr16-GFP–rich LDs (left) as well as Pdr16-GFP–poor LDs visualized by the neutral lipid dye MDH (right) in close proximity to the NVJ (marked by Nvj1-Cherry; black) and far from the NVJ (gray) were counted. *n* = 200 LDs for Pdr16-rich and Pdr16-poor LDs. **(C)** A collection of strains expressing all known LD proteins fused to either a C- or N-terminal GFP tag was crossed with either a strain expressing Erg6-Cherry, marking all LDs in the cell, or Pdr16-Cherry as an LD subpopulation marker. Displayed GFP-tagged proteins are enriched on a subset of Erg6-Cherry–positive LDs that are also enriched for Pdr16-Cherry. White arrows, LDs with enriched proteins; yellow arrows, other LDs. Bar, 5 µm.

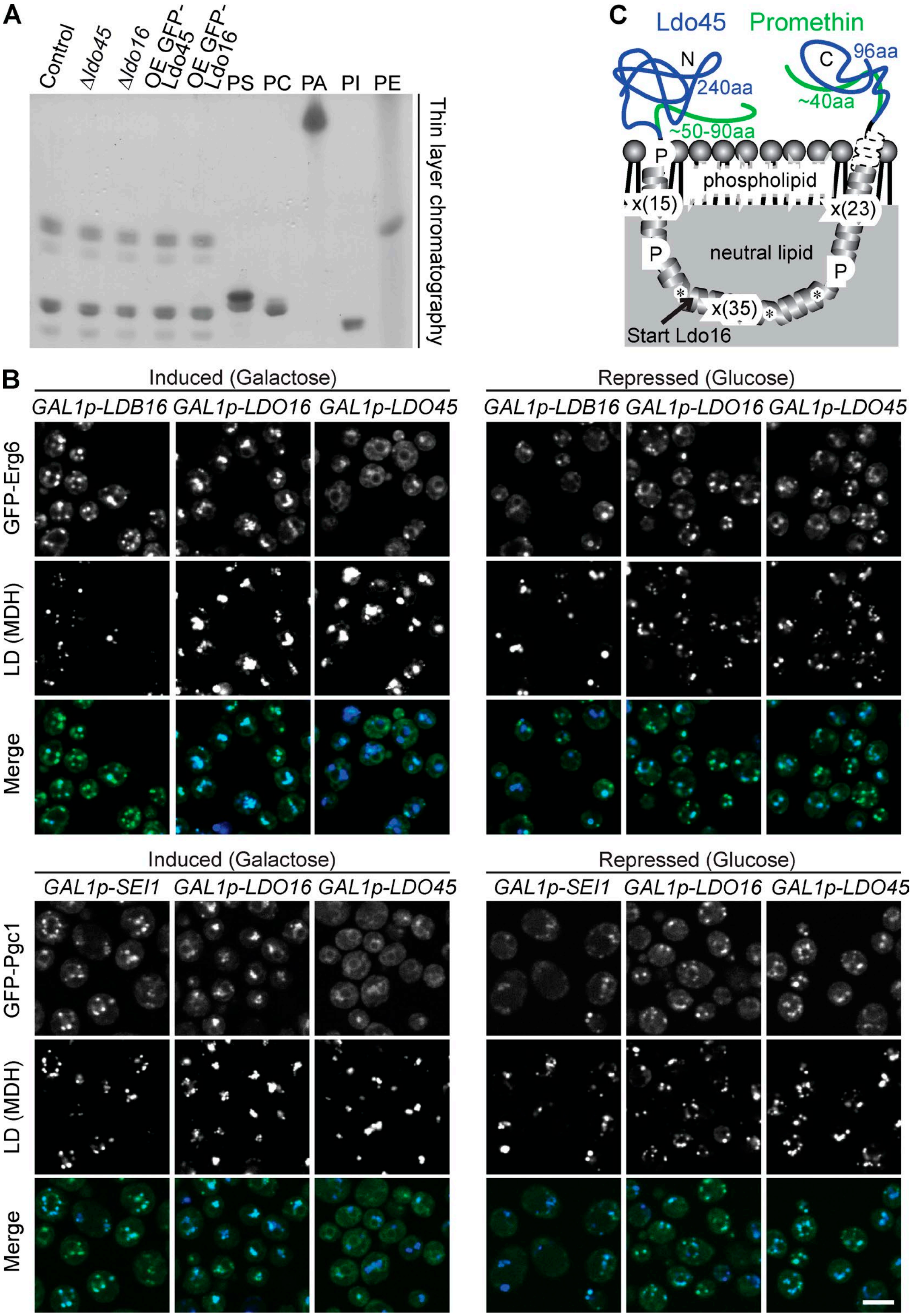

Figure S2. **Mechanistic aspects of Ldo functions. (A)** A crude LD fraction was isolated from indicated strains, and phospholipids were analyzed by thin-layer chromatography. PS, phosphatidylserine standard; PC, phosphatidylcholine; PA, phosphatidic acid; PI, phosphatidylinositol; PE, phosphatidylethanolamine. **(B)** Indicated strains were imaged under conditions of *GAL1p* repression (glucose) or overexpression (galactose), and LDs were stained with MDH. Both GFP-Erg6 and GFP-Pgc1 failed to properly localize to LDs upon Ldo45 overexpression as well as upon *LDB16* or *SEI1* repression. Bar, 5 µm. **(C)** Hypothetical model of Ldo45 from Saccharomycetes compared with the related protein promethin, found in 100 animal species (among them, humans) and >200 fungi. Both Ldo45 and promethin have highly hydrophilic N- and C-terminal cytoplasmic domains (Ldo45, blue; promethin, green), which bracket a hydrophobic helix ~75 residues long with the conserved motif P-X(15)-P-X(35)-P-X(~23). The prolines (P) and several glycines (represented by asterisks; 4 in Ldo45 and a mean of 5 in promethins) may induce kinks to turn the helix through 180°. Each individual X can be any residue, but overall, the helix is almost completely devoid of strongly hydrophilic residues and is rich in serine/threonine (~15%). The first residue of the Ldo16 protein is marked with an arrow. Hydrophobic helix domains are represented by solid lines; the hydrophilic helix has a dashed outline.

Provided online are three tables in Excel. Table S1 provides a list of hits from all screens performed in this study. Table S2 describes all strains used in this study. Table S3 shows all plasmids used in this study.
